# The Burden of Lip and Oral Cavity Cancer Among Women Across 204 Countries and Territories in the Context of the Framework Convention on Tobacco Control: An Interrupted Time Series Analysis

**DOI:** 10.3390/ijerph22101464

**Published:** 2025-09-23

**Authors:** Laila Menezes Hagen, Larissa Rodrigues Gasparini, Bruna Machado da Silva, Amanda Ramos da Cunha, Fernando Neves Hugo, José Miguel Amenábar

**Affiliations:** 1 Department of Stomatology, Federal University of Paraná, Curitiba 80210-170, Brazil; lailahagen@ufpr.br (L.M.H.); larissagasparini@ufpr.br (L.R.G.); bruna.machadodasilva@alumni.usp.br (B.M.d.S.); 2 Department of Epidemiology, School of Public Health, University of São Paulo, São Paulo 01246-904, Brazil; amandaracunha@usp.br; 3 Department of Epidemiology and Health Promotion, New York University College of Dentistry, New York, NY 10010, USA; fnh9064@nyu.edu

**Keywords:** women’s health, oral cancer, tobacco control, global health

## Abstract

Background: Historically, lip and oral cavity cancer (LOC) has been more prevalent among men, largely due to higher tobacco use in this group. However, over the past decades, smoking rates among women have risen and, in some regions, are approaching those of men. This shift highlights the urgent need to analyze the burden of LOC specifically in women, as they may respond differently to tobacco control policies. This study assessed whether the World Health Organization Framework Convention on Tobacco Control (WHO-FCTC), launched in 2003, and the implementation of MPOWER measures have influenced LOC trends among women. Methods: A controlled interrupted time series was conducted from 1990 to 2021, with the launch of the WHO-FCTC considered the intervention point. A total of 204 countries and territories were initially categorized into two groups: those without (G1) and with (G2) MPOWER coverage. G2 was further subdivided based on the median MPOWER score from 2007 to 2020 into G2A (equal to or below the median) and G2B (above the median). Analyses were also stratified by Socio-Demographic Index (SDI) levels. Female LOC rates were obtained from the Global Burden of Disease Study 2021. Prais-Winsten segmented regression was applied to estimate annual percent changes (APCs) in LOC rates before and after the WHO-FCTC. Results: Prior to the WHO-FCTC, most trends for G1 and G2A were stable, while all trends for G2B were increasing. After 2003, LOC rates increased across all groups, especially in G2B. In high-SDI settings, rising trends in G2B remained unchanged post-intervention, whereas G1 and G2A shifted from stable to increasing. Among low-SDI groups, slopes were mostly not statistically significant. Conclusions: These findings suggest that the WHO-FCTC has had no measurable impact on reducing LOC burden among women so far. Instead, rates have continued to rise in many regions, signaling a concerning trend for women’s global health.

## 1. Introduction

According to global estimates, lip and oral cavity cancer (LOC) currently constitutes the sixteenth most prevalent malignant neoplasm worldwide [1]. Historically, the burden of LOC has been considerably higher among men [2,3]; however, emerging evidence indicates a recent upward trend among women across several countries [4,5,6,7,8]. As with men, smoking and alcohol consumption are also identified as the primary known risk factors for this cancer among women [9,10]. Given the high mortality rates and the significant disabling potential associated with this malignancy [11], preventing LOC among women is critical to reducing its global burden.

The Framework Convention on Tobacco Control of the World Health Organization (WHO-FCTC) is the first global strategic instrument for addressing tobacco use. It is an international treaty that brings together a wide range of measures focused on reducing tobacco consumption, including actions to reduce the demand for products, combating illicit trade, as well as protection against involuntary exposure to tobacco smoke. Launched in 2003, the WHO-FCTC has been supported by the MPOWER measures since 2008, which were designed to strengthen the implementation of the treaty by signatory countries [12,13].

The WHO-FCTC may serve as a pivotal marker for the global reduction in the burden of tobacco-related diseases, including LOC. According to Silva et al. [14], following the launch of the WHO-FCTC, countries with stronger MPOWER scores have shown a decline in LOC trends compared to prior patterns. However, these results were reported for both sexes combined, leaving it unclear whether the LOC burden among women follows a similar trajectory, given the historically higher burden in men. Therefore, the aim of this study is to assess whether the WHO-FCTC has influenced LOC trends in women and whether stronger MPOWER measures have had an impact on these trends. Additionally, we examined whether countries with higher or lower Socio-demographic Index (SDI) levels exhibit distinct patterns.

## 2. Materials and Methods

This is a controlled interrupted time series, which evaluates the impact of a public intervention on a health outcome over time by comparing it to a control group that did not receive the intervention [15,16]. We followed the methodology developed by Silva et al. [14].

### 2.1. Data Collection

#### 2.1.1. MPOWER Scores

MPOWER is an acronym in which each letter represents a key component of tobacco control: monitoring tobacco use and prevention policies (M); protecting people from tobacco smoke (P); offer help to quit tobacco use (O); warn about the dangers of tobacco (W); enforce bans on tobacco advertising, promotion and sponsorship (E); and raise taxes on tobacco (R). Scores have been assigned to these six tobacco control measures, ranging from 1 to 5 for the ‘POWER’ measures and 1 to 4 for the ‘M’ measure. A score of 1 indicates either no data, no recent data, or no report, while higher scores reflect increased quality or intensity of the measures. Scores are assigned biennially [13]. The MPOWER scores were obtained from the Global Health Observatory on the WHO website [17]. For this study, MPOWER scores were collected from 2007 to 2020, except for the ‘R’ measure, which is only available from 2008 onwards. The six scores were summed by year for each country to form the MPOWER total.

#### 2.1.2. Lip and Oral Cavity Cancer Rates

The LOC rates were obtained from the GBD 2021 results tool [18]. The GBD cancer estimates are initially derived from population-based cancer registries and then refined through several sophisticated methodological steps, as detailed by the GBD 2019 Lip, Oral, and Pharyngeal Cancer Collaborators [2]. This process makes data available disaggregated by sex (or both sexes combined), cancer type, and age. For this study, data on females over the age of 20 were selected using the results tool. The metrics used were rates per 100,000 population, and the measures included were incidence, mortality, and disability-adjusted life years (DALYs), from 1990 to 2021.

#### 2.1.3. Socio-Demographic Index (SDI)

SDI is an average of the total fertility rate under the age of 25, lag-distributed income per capita, and mean education levels for those aged 15 and older, all of which can influence health outcomes. SDI ranges from 0 to 1, with lower values indicating worse social and economic conditions and higher values indicating better conditions [2]. SDI data for each country and territory by year (1990 to 2021) were retrieved from Global Health Data Exchange [19].

### 2.2. Study Design

The 204 countries and territories were first divided into two groups: G1—locations without MPOWER scores coverage (control group); and G2—locations with MPOWER scores coverage. Next, the average MPOWER score from 2007 to 2020 was calculated for each location of G2. The median of all countries’ averages was then determined and G2 was further divided into two subgroups: G2A—locations with an average MPOWER score equal to or lower than the overall median; and G2B—locations with an average MPOWER score higher than the overall median (Supplementary Figure S1) [14].

Additional analyses were conducted to examine how the effects varied between countries with higher and lower SDI levels. First, the average SDI from 1990 to 2021 was calculated for each location. Then, the median SDI across all 204 countries and territories was determined. Based on this, countries were divided into two groups: low-SDI—locations with an average SDI equal to or lower than the median; and high-SDI—locations with an average SDI higher than the median. Within each SDI group, subgroups G1, G2A, and G2B were also delineated (Supplementary Figure S2). The analysis was performed for the average annual incidence, mortality, and DALY rates for LOC for each of the three groups (G1, G2A, and G2B) [14].

### 2.3. Statistical Analysis

Segmented regression using the Prais-Winsten method was applied [20] to obtain the coefficients needed to calculate the annual percent change (APC) and its 95% confidence intervals (95% CI), as mathematically described by Antunes and Waldman [21]:

APC = (−1 + 10 ^β1^) × 100

APC ^lower^ = (−1 + 10 ^β1lower^) × 100

APC ^upper^ = (−1 + 10 ^β1upper^) × 100

where ^β1^ is the regression coefficient, and ^β1lower^ and ^β1upper^ are its 95% confidence interval bounds. This method allowed for the assessment of pre- and post-FCTC LOC trends. The post-WHO-FCTC period is represented by the slope of the time series following the launch of the WHO-FCTC in 2003. This slope reflects the post-FCTC trend while accounting for the pre-FCTC trajectory, thereby evaluating whether a gradual change in trend occurred after the intervention [15]. Consequently, two APCs were calculated for each series group—the trend and the slope—each of which could yield one of three possible outcomes:

- If the APC and its 95% CI are positive, the trend or slope is increasing.
- If the APC and its 95% CI are negative, the trend or slope is decreasing.
- If the 95% CI crosses zero, the trend or slope is considered stable, indicating no significant change.

The data were analyzed using Stata Software, version 13.1 (StataCorp, College Station, TX, USA).

## 3. Results

**Figure 1:**
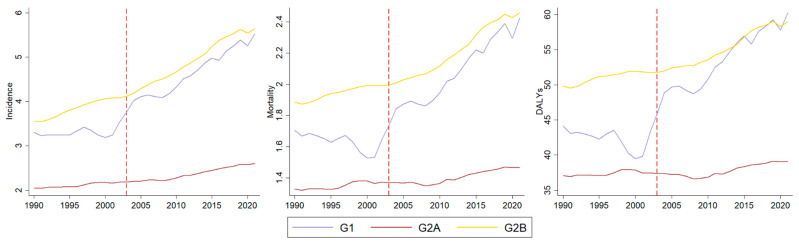
Incidence, mortality, and DALYs rates per 100,000 population for lip and oral cavity cancer before and after the launch of the WHO-FCTC (red dashed line) in 204 countries and territories groups.

Of the 204 countries and territories, 195 are covered by MPOWER measures (G2), while 9 are not (G1). The median MPOWER score across the 195 countries and territories covered was 19.67 (interquartile range [IQR]: 17–22.33), resulting in the composition of groups detailed in Supplementary Table S1, with G2A including 99 countries and territories and G2B including 96. Figure 1 illustrates the LOC rates among women over 20 years old for each group over time, showing an increase in rates across all groups after the launch of the WHO-FCTC. Table 1 presents the trends observed before the WHO-FCTC and indicates whether they remained unchanged or shifted (slopes) after the treaty. In the pre-FCTC period, the trends were stable for most indicators of G1 and G2A, while for G2B all indicators were increasing. Following WHO-FCTC launch, the slopes showed increases in all indicators for G1 and G2B. This indicates that G2B, the group with the highest MPOWER scores, exhibited increasing trends prior to the WHO-FCTC, and these trends accelerated even further following the treaty. G2A exhibited a significant increase only in incidence slope.

### Analysis by Socio-Demographic Index

**Figure 2:**
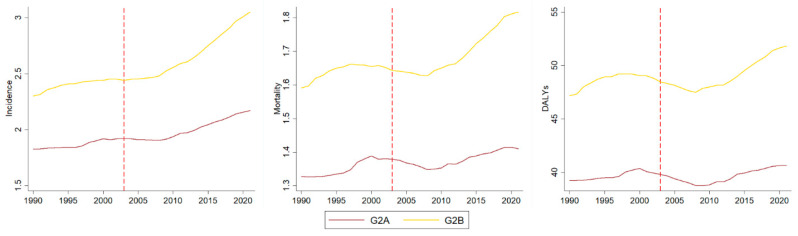
Incidence, mortality, and DALYs rates per 100,000 population for lip and oral cavity cancer before and after the launch of the WHO-FCTC (red dashed line) in low-SDI countries and territories groups.

The SDI median across all countries and territories was 0.587 (IQR: 0.422–0.732). The median MPOWER score of locations with an average SDI equal to or below this median (low-SDI) was 18 (IQR: 15.6–20.13). All countries in this group (*n* = 102) were covered by MPOWER scores, thus there is only G2 (A and B) in this analysis (group composition is shown in Supplementary Table S2), with G2A comprising 52 countries and territories and G2B comprising 50. The results of low-SDI countries are presented in Figure 2 and Table 2. In the period preceding the WHO-FCTC, incidence was increasing in both groups, while mortality increased in G2A. Overall, trends remained unchanged after the launch of the WHO-FCTC, with the only exception of an increasing incidence slope observed in group G2B.

**Figure 3:**
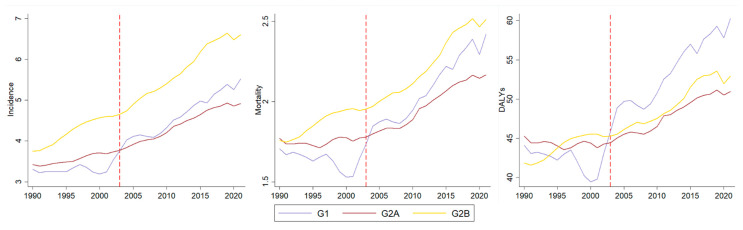
Incidence, mortality, and DALYs rates per 100,000 population for lip and oral cavity cancer before and after the launch of the WHO-FCTC (red dashed line) in high-SDI countries and territories groups.

Countries with an average SDI above 0.587, classified as high-SDI (*n* = 102), had a median MPOWER score of 22.07 (IQR: 19.6–23.47), resulting in G2A with 48 countries and territories and G2B with 45. The composition of groups is detailed in Supplementary Table S3. The slopes for G1 (*n* = 9) and G2A indicated increases relative to their respective pre-FCTC trends, which had been mostly stable. For G2B, no changes in the increasing trends seen in the pre-FCTC period were observed (Figure 3 and Table 3).

## 4. Discussion

Previous reports have found the effects of WHO-FCTC and high MPOWER scores on tobacco use globally [12,22,23,24]. It is estimated that approximately 1.2 billion smokers were affected by MPOWER policies between 2007 and 2020, potentially resulting in a reduction of more than 28 million smoking-attributable deaths [23]. As a result of this scenario, a reduction in the burden of LOC is also anticipated. That is what the study by Silva et al. [14] found: LOC trends post-FCTC are modifying and in decline for both sexes together among countries with stronger MPOWER scores for low- and high-SDI countries. However, one remaining question is whether these findings hold true specifically for women. In this interrupted time series analysis, we applied the same methodology used by Silva et al., with the difference that the analysis focused on the female population and was based on GBD 2021. Unlike the earlier findings, this study did not observe a shift toward a declining trend in LOC rates among women following the launch of the WHO-FCTC, regardless of MPOWER score levels and SDI levels. Moreover, the group with the highest MPOWER scores exhibited increasing trends both before and after the WHO-FCTC, a concerning scenario suggesting that the treaty has not yet had any observable impact on LOC among women.

Globally, the burden of LOC is higher in men than in women [2,25,26,27]. This pattern mirrors the historically higher prevalence of smoking among men [28]. However, while smoking prevalence among men peaked earlier, the peak among women occurred a few decades later [29,30]. This delayed pattern may help explain the more recent global increases in some tobacco-related diseases among women, including lung cancer [31,32] and oral cancer [2,33]. The impact of WHO-FCTC on women may not yet be reflected in the burden of some tobacco-related diseases and could only become apparent in the coming years. This is supported by the fact that between 1990 and 2019, the smoke prevalence age-standardized declined in only 68 countries (33%) among women in contrast with 135 (66%) countries that declined among men. However, after the WHO-FCTC ratification, smoking prevalence among women declined faster in 136 countries (67%) [28]. Furthermore, according to Flor et al. [22], between 2009 and 2017, smoking prevalence declined by 7.7% among men and 15.2% among women, suggesting that the greatest decline among women occurred more recently.

Despite that, some studies support the idea that women may be less responsive to tobacco control policies than men [22,24]. For instance, P measure score was not associated with reductions in smoking prevalence among women aged 15–29 or those over 50. Similarly, reduced tobacco accessibility did not correlate with lower smoking prevalence among women aged 50 and older [22]. Ngo et al. [24] dealt with the composed MPOWER scores and found that an increase in 1 score was associated with a reduction of 0.8% in smoke prevalence between men and adults, but no association was found for women.

Women may face greater challenges in quitting smoking compared to men [34,35]. This is partly due to women exhibiting stronger behavioral dependence on smoking, whereas men tend to show greater nicotine dependence. This affects the efficacy of smoking cessation therapies; for instance, nicotine replacement therapy has been shown to be less effective in women than in men [36]. Additionally, the menstrual cycle phase may influence women’s withdrawal symptoms and nicotine’s effects [37,38]. Other factors contributing to sex differences in smoking cessation include fear of weight gain—particularly among young women [39,40,41]. Moreover, women appear to experience stress as a more significant barrier to quitting than men, identifying coercion as an additional obstacle [42]. Thus, analyzing tobacco use and control through the lens of sex and gender is essential.

Another perspective to look at our findings is that women may have a smaller fraction of LOC burden attributed to smoking. It was observed that only 11.4% of LOC deaths among women were attributable to smoking, in contrast to 42.3% of men [2]. On the other hand, chewing tobacco is responsible for 27.6% of LOC deaths among women, while in men it is responsible for 14.1% [2]. Smokeless tobacco (ST) use is associated with oral cancer, with over 85% of the global ST burden concentrated in South and Southeast Asia, and India accounting for most of it [43]. The population attributable fraction (PAF) of oral cancer due to ST or areca nut consumption among women is notably high in countries such as Papua New Guinea (83.8%), Bangladesh (60.8%), and India (51.5%) [44]. Although ST falls within the scope of the WHO-FCTC and has been targeted by tobacco control policies in many countries [45], stronger global efforts are needed to expand the inclusion of these products in tobacco control measures [46]. Moreover, areca nut use continues to be a risk factor requiring urgent attention in oral cancer prevention, particularly across South and Southeast Asia and several Oceania countries [47].

Among most of the countries, prevalence of ST use is not high [43,48]. In these countries, smoking and/or other risk factors—such as alcohol consumption, low intake of fruits and vegetables, and low socioeconomic status [49]—may still play a significant role in increasing the burden of LOC among women. Additionally, unknown risk factors may be contributing to this scenario. Previous studies have suggested that a proportion of oral cancer cases are not attributable to tobacco or alcohol use [50], although the alternative pathways for LOC development remain unclear.

Given the complexities outlined above, the issues surrounding smoking cessation and the burden of LOC among women are multifaceted. Recent attention to sex and gender may help illuminate the distinct meanings of smoking within women’s health and social contexts. Such an understanding is crucial for guiding future directions in tobacco control among women, as it can inform more effective cessation strategies. Key considerations for interventions targeting smoking in women include addressing biological and psychosocial factors, identifying and overcoming barriers to change, providing appropriate support, and integrating social justice perspectives, thereby offering a holistic view of the broader context of a woman’s life [51]. From a broader perspective, tobacco control policies can also promote gender equality by reducing women’s vulnerability to smoking without reinforcing paternalistic ideals [52]. In general, these policies should aim to include and empower women in cessation efforts, rather than creating exclusionary effects.

### Limitations

The influence of other risk factors on LOC distribution is considered one limitation of this study, as we only analyzed one risk factor intervention. Furthermore, our main limitation is the uncertainty surrounding the time lag between the implementation of tobacco control policies and their measurable impact on the burden of LOC. As previously noted, the peak in smoking prevalence among women occurred later than among men [29], which may lead to a delayed impact of tobacco control measures on smoking prevalence among women. Considering that, the LOC burden on women should continue to be explored through time series to understand if this is a part of the delay. Finally, the small number of countries without MPOWER coverage is another important limitation for this study, as one outlier could weigh on the results for this group.

## 5. Conclusions

No reductions in the burden of LOC among women have been observed following the implementation of the WHO-FCTC, regardless of the strength of MPOWER measures or countries’ SDI levels. On the contrary, the rising burden of LOC is particularly alarming for women’s health. These findings underscore the urgent need for further research into the effectiveness of tobacco control policies in addressing tobacco-related diseases among women.

## Figures and Tables

**Table 1:** Incidence, mortality and DALYs trends and slopes of lip and oral cavity cancer in 204 countries and territories groups.

APC	Group	Incidence [95% CI]		Mortality [95% CI]		DALYs [95% CI]	
Trend	G1	0.78 [−0.07 ± 1.64]	Stability	−0.14 [−0.86 ± 0.58]	Stability	0.01 [−0.90 ± 0.94]	Stability
	G2A	**0.48 [0.19 ± 0.77]**	**Increasing**	0.22 [−0.08 ± 0.51]	Stability	0.05 [−0.23 ± 0.33]	Stability
	G2B	**1.23 [0.92 ± 1.54]**	**Increasing**	**0.42 [0.12 ± 0.72]**	**Increasing**	**0.28 [0.03 ± 0.53]**	**Increasing**
Slope	G1	**1.65 [0.42 ± 2.91]**	**Increasing**	**2.26 [1.20 ± 3.33]**	**Increasing**	**1.81 [0.46 ± 3.18]**	**Increasing**
	G2A	**0.48 [0.06 ± 0.90]**	**Increasing**	0.16 [−0.26 ± 0.58]	Stability	0.18 [−0.22 ± 0.59]	Stability
	G2B	**0.50 [0.05 ± 0.95]**	**Increasing**	**0.71 [0.29 ± 1.14]**	**Increasing**	**0.43 [0.06 ± 0.79]**	**Increasing**

Bold indicates statistically significant trends.

**Table 2:** Incidence, mortality and DALYs trends and slopes of lip and oral cavity cancer in low-SDI countries and territories groups.

APC	Group	Incidence [95% CI]		Mortality [95% CI]		DALYs [95% CI]	
Trend	G2A	**0.38 [0.08 ± 0.69]**	**Increasing**	**0.30 [0.06 ± 0.53]**	**Increasing**	0.13 [−0.12 ± 0.38]	Stability
	G2B	**0.51 [0.14 ± 0.88]**	**Increasing**	0.30 [−0.02 ± 0.62]	Stability	0.27 [−0.05 ± 0.59]	Stability
Slope	G2A	0.28 [−0.15 ± 0.70]	Stability	−0.17 [−0.51 ± 0.16]	Stability	−0.04 [−0.38 ± 0.30]	Stability
	G2B	**0.64 [0.14 ± 1.15]**	**Increasing**	0.20 [−0.24 ± 0.63]	Stability	0.04 [−0.39 ± 0.48]	Stability

Bold indicates statistically significant trends.

**Table 3:** Incidence, mortality and DALYs trends and slopes of lip and oral cavity cancer in high-SDI countries and territories groups.

APC	Group	Incidence [95% CI]		Mortality [95% CI]		DALYs [95% CI]	
Trend	G1	0.78 [−0.07 ± 1.64]	Stability	−0.14 [−0.86 ± 0.58]	Stability	0.01 [−0.90 ± 0.94]	Stability
	G2A	**0.83 [0.55 ± 1.11]**	**Increasing**	0.01 [−0.26 ± 0.28]	Stability	−0.14 [−0.33 ± 0.05]	Stability
	G2B	**1.82 [1.36 ± 2.28]**	**Increasing**	**0.84 [0.43 ± 1.25]**	**Increasing**	**0.65 [0.28 ± 1.03]**	**Increasing**
Slope	G1	**1.65 [0.42 ± 2.91]**	**Increasing**	**2.26 [1.20 ± 3.33]**	**Increasing**	**1.81 [0.46 ± 3.18]**	**Increasing**
	G2A	**0.72 [0.32 ± 1.13]**	**Increasing**	**1.16 [0.77 ± 1.56]**	**Increasing**	**0.97 [0.70 ± 1.25]**	**Increasing**
	G2B	0.11 [−0.55 ± 0.76]	Stability	0.53 [−0.06 ± 1.13]	Stability	0.19 [−0.34 ± 0.73]	Stability

Bold indicates statistically significant trends.

## Data Availability

The data used in this study are estimates resulting from the 2021 GBD study. They are available on the website of the World Health Organization [17] and the Institute for Health Metrics and Evaluation [18,19] and can be publicly consulted and downloaded.

## Supplementary Materials

The following supporting information can be downloaded at: https://www.mdpi.com/article/10.3390/ijerph22101464/s1, Figure S1: Classification of countries and territories based on MPOWER coverage and the overall MPOWER median, resulting in three groups analyzed (G1, G2A, and G2B); Figure S2: Classification of countries and territories first by the median socio-demographic index (SDI), and then by MPOWER coverage and the MPOWER median within each SDI group; Table S1: Group composition for the overall analysis, categorized based on MPOWER coverage and the median MPOWER score across 204 countries and territories; Table S2: Group composition for the low-SDI countries and territories, based on their median MPOWER score; Table S3: Group composition for the high-SDI countries and territories, based on their median MPOWER score.

## Abbreviations

The following abbreviations are used in this manuscript:

LOC	Lip and oral cavity cancer
WHO-FCTC	World Health Organization Framework Convention on Tobacco Control
DALYs	Disability-adjusted life years
SDI	Socio-demographic Index
APC	Annual Percent Change
95% CI	95% Confidence Interval
IQR	Interquartile range
ST	Smokeless tobacco
PAF	Population attributable fraction
