# Supplementary material for: The Burden of Lip and Oral Cavity Cancer Among Women Across 204 Countries and Territories in the Context of the Framework Convention on Tobacco Control: An Interrupted Time Series Analysis
Source: Int J Environ Res Public Health. 2025 Sep 23;22(10):1464. doi: 10.3390/ijerph22101464 (PMC12563127; doi:10.3390/ijerph22101464)
Supplement: Supplementary file 1 [file ijerph-22-01464-s001.zip › ijerph-3862679-supplementary.pdf]

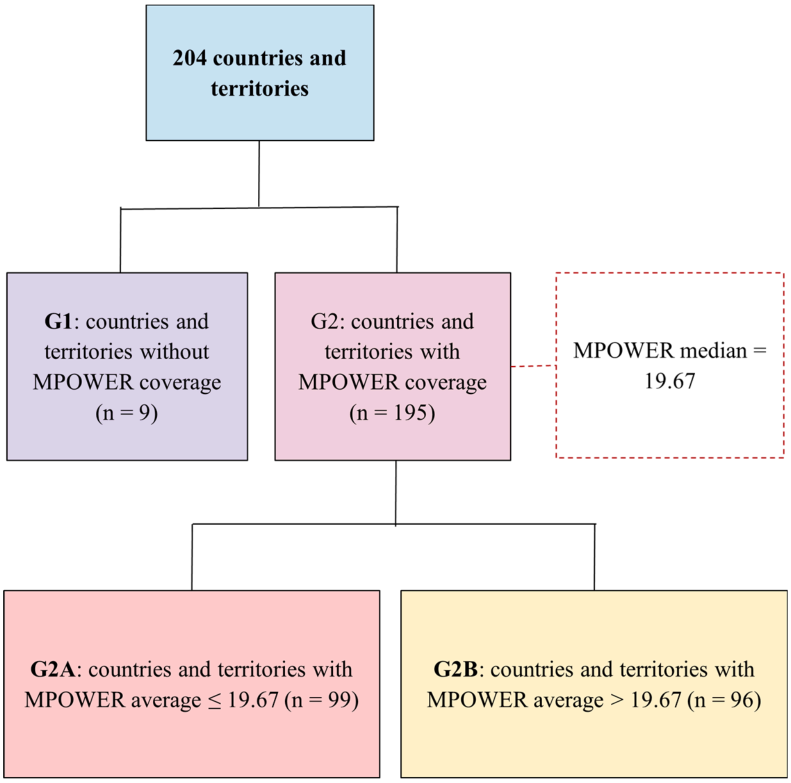

Figure S1. Classification of countries and territories based on MPOWER coverage and the overall MPOWER median, resulting in three groups analyzed (G1, G2A, and G2B).

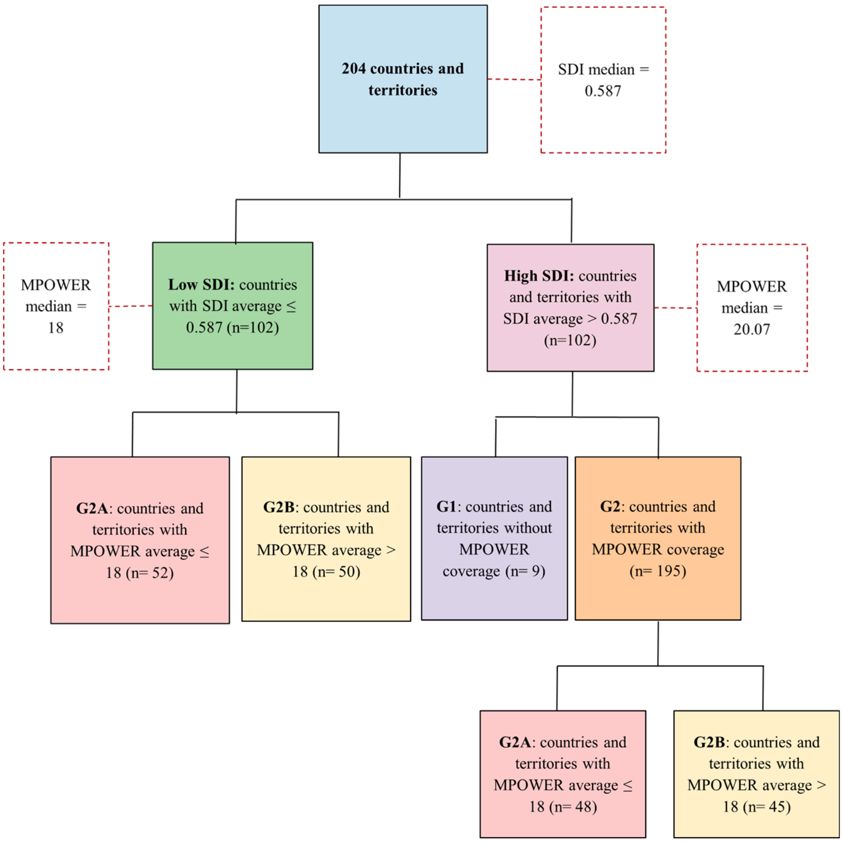

Figure S2. Classification of countries and territories first by the median socio-demographic index (SDI), and then by MPOWER coverage and the MPOWER median within each SDI group.

Table S1. Group composition for the overall analysis, categorized based on MPOWER coverage and the median MPOWER score across 204 countries and territories.

GROUP/ SUBGROUP		COUNTRIES AND TERRITORIES
**G1 (n=9)**		American Samoa, Bermuda, Greenland, Guam, Northern Mariana Islands, Puerto Rico, Taiwan (Province of China), Tokelau, United States Virgin Islands.
**G2 (n=195)**	**A* (n=99)**	Afghanistan, Algeria, Andorra, Angola, Antigua and Barbuda, Armenia, Azerbaijan, Bahamas, Bahrain, Barbados, Belarus, Belize, Benin, Bhutan, Bosnia and Herzegovina, Botswana, Burkina Faso, Burundi, Cabo Verde, Cambodia, Cameroon, Central African Republic, China, Comoros, Congo, Cote d'Ivoire, Cuba, Democratic People's Republic of Korea, Democratic Republic of the Congo, Dominica, Dominican Republic, Equatorial Guinea, Eritrea, Eswatini, Ethiopia, Fiji, Gabon, Gambia, Ghana, Grenada, Guatemala, Guinea, Guinea-Bissau, Guyana, Haiti, Indonesia, Iraq, Israel, Jamaica, Japan, Kiribati, Lao People's Democratic Republic, Lesotho, Liberia, Libya, Malawi, Maldives, Mali, Marshall Islands, Mauritania, Mexico, Micronesia (Federated States of), Monaco, Morocco, Mozambique, Nauru, Nicaragua, Niger, Nigeria, Niue, Oman, Papua New Guinea, Paraguay, Rwanda, Saint Kitts and Nevis, Saint Lucia, Saint Vincent and the Grenadines, San Marino, Sao Tome and Principe, Senegal, Sierra Leone, Solomon Islands, Somalia, South Africa, South Sudan, Sudan, Syrian Arab Republic, Tajikistan, Timor-Leste, Togo, Trinidad and Tobago, Tunisia, Tuvalu, Uganda, United Republic of Tanzania, United States of America, Uzbekistan, Zambia, Zimbabwe.
	**B (n=96)**	Albania, Argentina, Australia, Austria, Bangladesh, Belgium, Bolivia (Plurinational State of), Brazil, Brunei Darussalam, Bulgaria, Canada, Chad, Chile, Colombia, Cook Islands, Costa Rica, Croatia, Cyprus, Czechia, Denmark, Djibouti, Ecuador, Egypt, El Salvador, Estonia, Finland, France, Georgia, Germany, Greece, Honduras, Hungary, Iceland, India, Iran (Islamic Republic of), Ireland, Italy, Jordan, Kazakhstan, Kenya, Kuwait, Kyrgyzstan, Latvia, Lebanon, Lithuania, Luxembourg, Madagascar, Malaysia, Malta, Mauritius, Mongolia, Montenegro, Myanmar, Namibia, Nepal, Netherlands (Kingdom of the), New Zealand, North Macedonia, Norway, Pakistan, Palau, Panama, Peru, Philippines, Poland, Portugal, Qatar, Republic of Korea, Republic of Moldova, Romania, Russian Federation, Samoa, Saudi Arabia, Serbia, Seychelles, Singapore, Slovakia, Slovenia, Spain, Sri Lanka, Suriname, Sweden, Switzerland, Thailand, Tonga, Turkmenistan, Türkiye, Ukraine, United Arab Emirates, United Kingdom of Great Britain and Northern Ireland, Uruguay, Vanuatu, Venezuela (Bolivarian Republic of), Viet Nam, Yemen, occupied Palestinian territory, including east Jerusalem.

*Armenia, Belarus and Jamaica had an average MPOWER score equal to the overall median, resulting in a total of 99 countries in G2A.

Table S2. Group composition for the low-SDI countries and territories, based on their median MPOWER score.

GROUP*/ SUBGROUP		COUNTRIES AND TERRITORIES
**G2 (n=102)**	**A** (n=52)**	Afghanistan, Algeria, Angola, Belize, Bhutan, Botswana, Burundi, Cabo Verde, Central African Republic, Comoros, Côte d'Ivoire, Democratic People's Republic of Korea, Democratic Republic of the Congo, Dominican Republic, Equatorial Guinea, Eritrea, Eswatini, Ethiopia, Gabon, Gambia, Ghana, Grenada, Guinea, Guinea-Bissau, Guyana, Haiti, Lesotho, Liberia, Malawi, Mali, Mauritania, Micronesia (Federated States of), Mozambique, Nicaragua, Nigeria, Papua New Guinea, Paraguay, Rwanda, Saint Vincent and the Grenadines, Sao Tome and Principe, Sierra Leone, Somalia, South Sudan, Sudan, Syrian Arab Republic, Tajikistan, Timor-Leste, Togo, United Republic of Tanzania, Uzbekistan, Zambia, Zimbabwe.
	**B (n=50)**	Bangladesh, Benin, Bolivia (Plurinational State of), Brazil, Burkina Faso, Cambodia, Cameroon, Chad, Colombia, Congo, Djibouti, Ecuador, Egypt, El Salvador, Guatemala, Honduras, India, Indonesia, Iraq, Kenya, Kiribati, Kyrgyzstan, Lao People's Democratic Republic, Madagascar, Maldives, Marshall Islands, Mexico, Mongolia, Morocco, Myanmar, Namibia, Nauru, Nepal, Niger, Pakistan, Peru, Philippines, Samoa, Senegal, Solomon Islands, Suriname, Tonga, Tuvalu, Türkiye, Uganda, Vanuatu, Venezuela (Bolivarian Republic of), Viet Nam, Yemen, occupied Palestinian territory, including east Jerusalem.

* In this group there are no countries without MPOWER coverage.

**In this group, Ghana e Papua New Guinea had an average MPOWER score equal to the median, resulting in a total of 52 countries in G2A.

Table S3. Group composition for the high-SDI countries and territories, based on their median MPOWER score.

GROUP/ SUBGROUP		COUNTRIES AND TERRITORIES
**G1 (n=9)**		American Samoa, Bermuda, Greenland, Guam, Northern Mariana Islands, Puerto Rico, Taiwan (Province of China), Tokelau, United States Virgin Islands.
**G2 (n=93)**	**A* (n=48)**	Andorra, Antigua and Barbuda, Armenia, Austria, Azerbaijan, Bahamas, Bahrain, Barbados, Belarus, Bosnia and Herzegovina, Brunei Darussalam, China, Cuba, Dominica, Fiji, Georgia, Germany, Iceland, Israel, Jamaica, Japan, Jordan, Kazakhstan, Kuwait, Lebanon, Libya, Luxembourg, Monaco, Montenegro, Netherlands (Kingdom of the), Niue, Oman, Palau, Qatar, Republic of Korea, Russian Federation, Saint Kitts and Nevis, Saint Lucia, San Marino, Saudi Arabia, South Africa, Sweden, Switzerland, Trinidad and Tobago, Tunisia, Turkmenistan, United Arab Emirates, United States of America.
	**B (n=45)**	Albania, Argentina, Australia, Belgium, Bulgaria, Canada, Chile, Cook Islands, Costa Rica, Croatia, Cyprus, Czechia, Denmark, Estonia, Finland, France, Greece, Hungary, Iran (Islamic Republic of), Ireland, Italy, Latvia, Lithuania, Malaysia, Malta, Mauritius, New Zealand, North Macedonia, Norway, Panama, Poland, Portugal, Republic of Moldova, Romania, Serbia, Seychelles, Singapore, Slovakia, Slovenia, Spain, Sri Lanka, Thailand, Ukraine, United Kingdom of Great Britain and Northern Ireland, Uruguay.

*Austria, Jordan and Kazakhstan had an average MPOWER score equal to the median, resulting in a total of 48 countries in G2A.
